# Novel function of the tumor suppressor PML at ER-mitochondria sites in the control of autophagy

**DOI:** 10.18632/oncotarget.18974

**Published:** 2017-07-04

**Authors:** Sonia Missiroli, Massimo Bonora, Simone Patergnani, Carlotta Giorgi

**Affiliations:** Carlotta Giorgi: Department of Morphology, Surgery and Experimental Medicine, Section of Pathology, Oncology and Experimental Biology, Laboratory for Technologies of Advanced Therapies, University of Ferrara, Ferrara, Italy

**Keywords:** autophagy, cancer, PML

Autophagy is a tightly regulated lysosomal degradation process that mediates the sequestration of intracellular entities to form autophagosomes, and their delivery to lysosomes for bulk degradation [1].

Autophagy plays a key role in the maintenance of cellular homeostasis, in baseline conditions as well as in response to stress. It can function as either a pro-death or pro-survival mechanism in cancer cells, depending on the different stage of tumor development and the cell type [1]. During tumor initiation autophagy has preventive effects; nevertheless once malignant transformation has occurred, autophagy is believed to promote tumor progression, conferring stress resistance and thereby maintaining cancer cell survival [1].

Cellular energetics represents a critical regulatory point for autophagy. Indeed, 5’ adenosine monophosphate-activated protein kinase (AMPK) senses the equilibrium between the phosphorylation status of the adenine nucleotide pool. When equilibrium is shifted to low phosphorylation of nucleotides (especially AMP), AMPK is activated and promotes autophagy to recycle energetic constituents. Mitochondria are a major site of production of higher energy state adenine nucleotide [2]. The high respiratory status of mitochondria allow low AMPK activity, thus maintaining autophagy at low levels [2]. Mitochondria participate in intracellular signaling by different routes. The most characterized sites are the contact sites between mitochondria and endoplasmic reticulum (ER), termed mitochondria-ER associated membranes (MAMs), which provide a platform for the regulation of different processes [3].

This signaling platform participates in the regulation of autophagy. Ca^2+^ transfer between ER and mitochondria supports mitochondrial respiration and then suppresses autophagy through low AMPK activity [3].

Additionally, the ER-mitochondria interface contributes to autophagosome formation, and proteins localized at MAM compartments are indispensable for proper autophagic vesicle formation [4].

Several oncogenes and oncosuppressors localize at MAMs to regulate communication between organelles and alter cell proliferation or sensitivity to cell death stimuli [3].

Taken together, these observations led us to hypothesize about the possible involvement of promyelocytic leukemia protein (PML) in autophagic process [5]. PML is a tumor suppressor gene that was originally identified at the breakpoint of the t(15;17) translocation of acute promyelocytic leukemia (APL) and is frequently lost or exhibits aberrant expression in human solid tumors and hematopoietic malignancies. We recently identified that PML localizes to the ER and MAMs, where it regulates Ca^2+^-dependent apoptosis by blocking the activity of IP3R3 and promoting the formation of a multiprotein complex containing IP3R3, AKT and the protein phosphatase PP2a [6]. In our study, we provide evidence that PML at MAMs also plays an essential role in the control of autophagy. At these sites, PML represses autophagosome formation, as detected in live imaging experiments and via immunoblotting, and thus autophagic induction.

We observed increased abundance of LC3-II in *Pml*^*-/-*^ mouse embryonic fibroblasts (MEFs) and in the livers and skeletal muscles of adult *Pml*^*-/-*^ mice compared with WT conditions; moreover, loss of PML increased autophagosome biogenesis.

In particular, PML localization at MAMs is necessary for repressing autophagy. In fact, an erPML construct (which contains the entire PML protein targeted to the outer surface of the ER) suppressed the elevated levels of autophagy when re-introduced into *Pml*^*-/-*^ MEFs. In contrast, a control construct targeted to nuclei failed. Given the tight interaction between PML and p53 and that p53 localizes at ER/MAMs [7], we sought to investigate the possible role of PML in autophagy in relation to p53. Through subcellular fractionation, we clearly demonstrate that p53 operates as a bridge to maintain correct PML localization at MAMs, which is fundamental for the repression of autophagy.

What are the molecular mechanisms involved in PML-dependent autophagy regulation? We examined the relationship between PML and a multiprotein complex that includes AMP-activated protein kinase (AMPK), mammalian target of rapamycin (mTOR) and unc-51-like kinase 1 (Ulk1) that is emerging as an important player in the regulation of autophagy.

Moreover, we speculate that downregulated ER-mitochondria Ca^2+^ transfer is important for induction of autophagy given the new critical role of Ca^2+^ in the formation of autophagosomes.

In the absence of PML, the release of Ca^2+^ from the ER into the mitochondria and the production of ATP are reduced [6].

Our results demonstrated that *Pml*^*-/-*^ cells and tissues exhibited increased AMPK phosphorylation levels, Ulk1 hyper-phosphorylation and reduced phosphorylation of mTOR and its substrate p70^S6K^, leading to increased autophagy. Interestingly, restoration of Ca^2+^ signaling in a *Pml*^*-/-*^ background by overexpressing mitochondrial Ca^2+^ uniporter (MCU) restores basal levels of autophagy.

These data indicate that the loss of PML from MAMs confers an alternative pathway to sustain mitochondrial activity and thus cell survival during stress conditions, promoting cell survival in the tumor environment.

**Figure 1:**
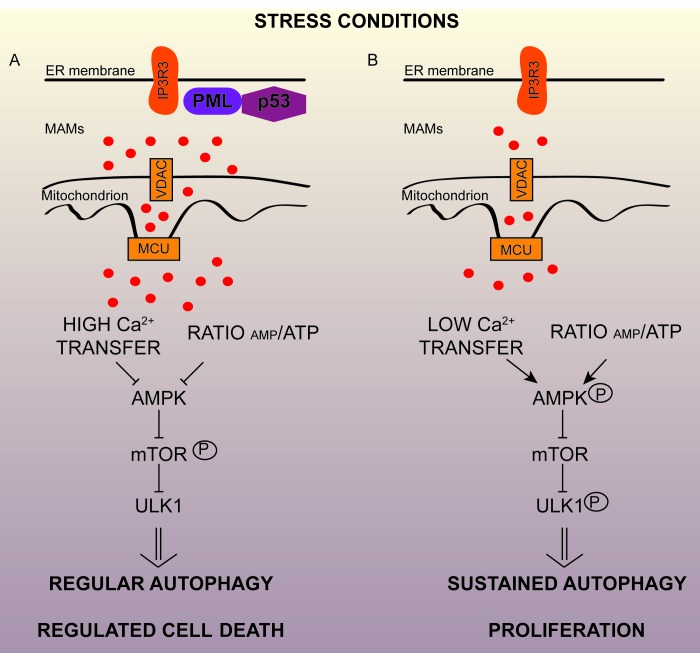
Extranuclear promyelocytic leukemia (PML) regulates autophagy and cancer progression from mitochondria-associated ER membranes (MAMs) **A.** In stress conditions (nutrient deprivation or the administration of chemotherapeutic agents) PML and p53 at endoplasmic reticulum/MAM contact sites are necessary for modulating autophagy and for promoting regulated cell death. **B.** Conversely, PML- or p53-deficient cancer cells use autophagy as a cell survival strategy during stress conditions, so autophagy may represent an important mechanism of resistance to cancer treatments. Loss of PML reduces IP3R-mediated Ca2+ transfer from the ER to mitochondria, that results in the activation of AMPK, which activates pro-survival autophagy by a mechanism involving mTOR/Ulk-1 pathways, allowing cellular proliferation and tumor maintenance. AMPK, AMP-activated protein kinase; mTORC1, mechanistic target of rapamycin complex 1; ULK1, unc-51-like autophagy-activating kinase 1.

We further investigated the cross-talk between autophagy and PML-related cell death in *in vivo* models. We observed that the absence of PML promotes tumor development associated with resistance to anticancer drugs due to increased autophagy levels in the tumor. Pharmacological inhibition of autophagy through chloroquine administration restored chemotherapy-related apoptosis (Figure 1).

Our findings hold therapeutic implications for the treatment of solid tumors associated with PML down-regulation and allow for further consideration for the MAM microenvironment that could be a fundamental background for the regulation of autophagic-dependent cancers.
